# Supplementary material for: Impact of Oncogenic Changes in p53 and KRAS on Macropinocytosis and Ferroptosis in Colon Cancer Cells and Anticancer Efficacy of Niclosamide with Differential Effects on These Two Processes
Source: Cells. 2024 May 30;13(11):951. doi: 10.3390/cells13110951 (PMC11171492; doi:10.3390/cells13110951)
Supplement: Supplementary file 1 [file cells-13-00951-s001.zip › cells-2966804-supplementary.pdf]

Supplemental Materials

Supplementary Table S1: Primer sequences

Gene	Forward	Reverse
SLC38A5	GTTGGGGCCATGTCCAGTTA	AGTGTTTCATGAGGGCGAGG
SLC38A3	GAGGCCAGACATCTGACTGTT	GGTCCTCGACCCTCTGGTT
SLC7A11	TGTGTGGGGTCCTGTCACTA	CAGTAGCTGCAGGGCGTATT
SLC3A2	CTCGTGGTTCTCCACTCAGG	CCGCAATCAAGAGCCTGTCT
18S	CCCGTTGAACCCCATTCGT	GCCTCACTAAACCATCCAATCGGTA
SLC38A5-p53 promoter	ACTAGGACTTTGCTGCCCAG	ACAGGCAGAGCACTTAAGACA
SLC38A5-p53 promoter (negative control)	GCCATTTGCTGAAATGC	AACTTCCAGGTTCAAGCCCT
SLC38A5-MYC promoter	GAGGCCTCTTGTCCACCTG	TTGTCCAAGCCCTGAGAAGT

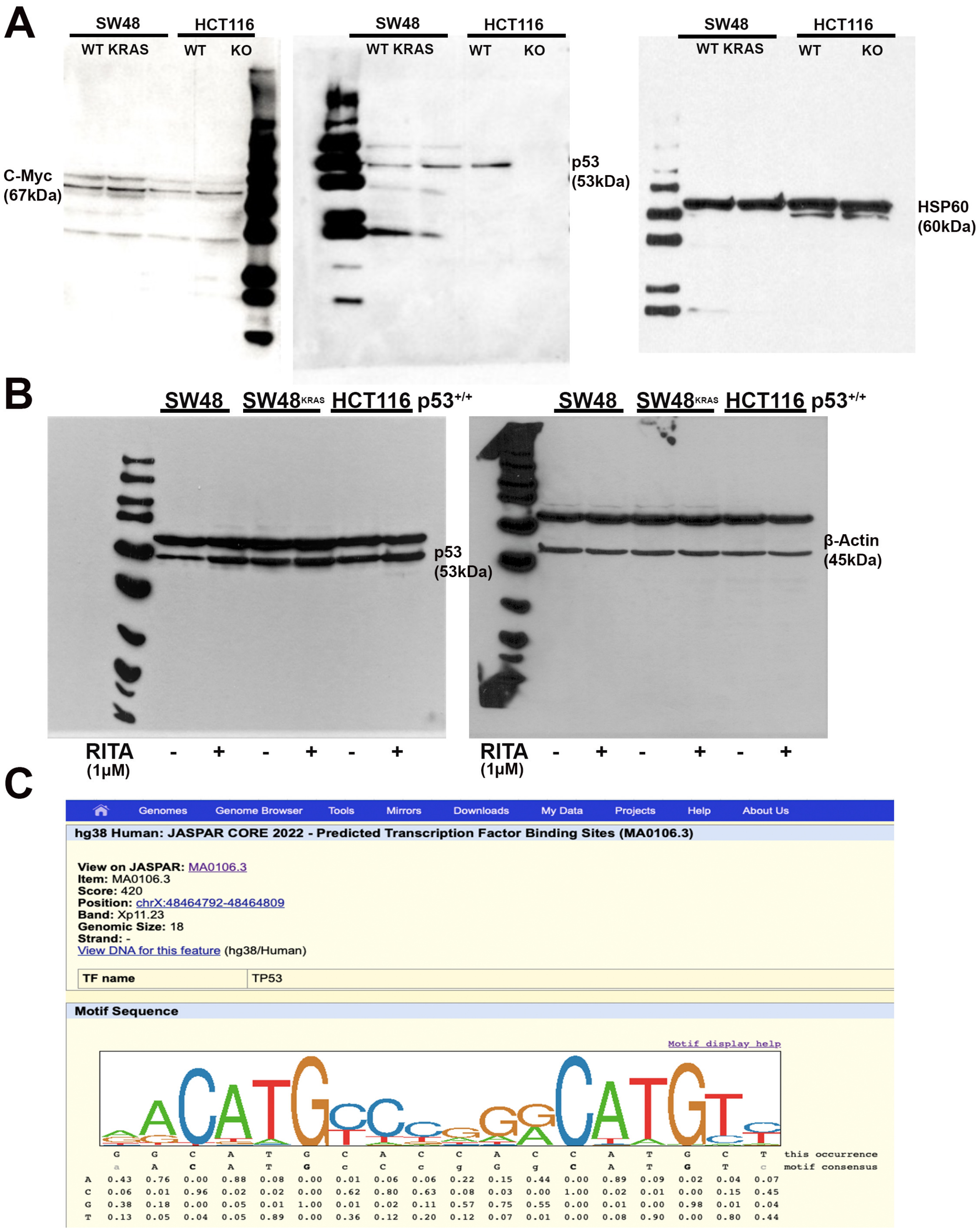

Supplementary Figure S1. Original blot for Fig 2.

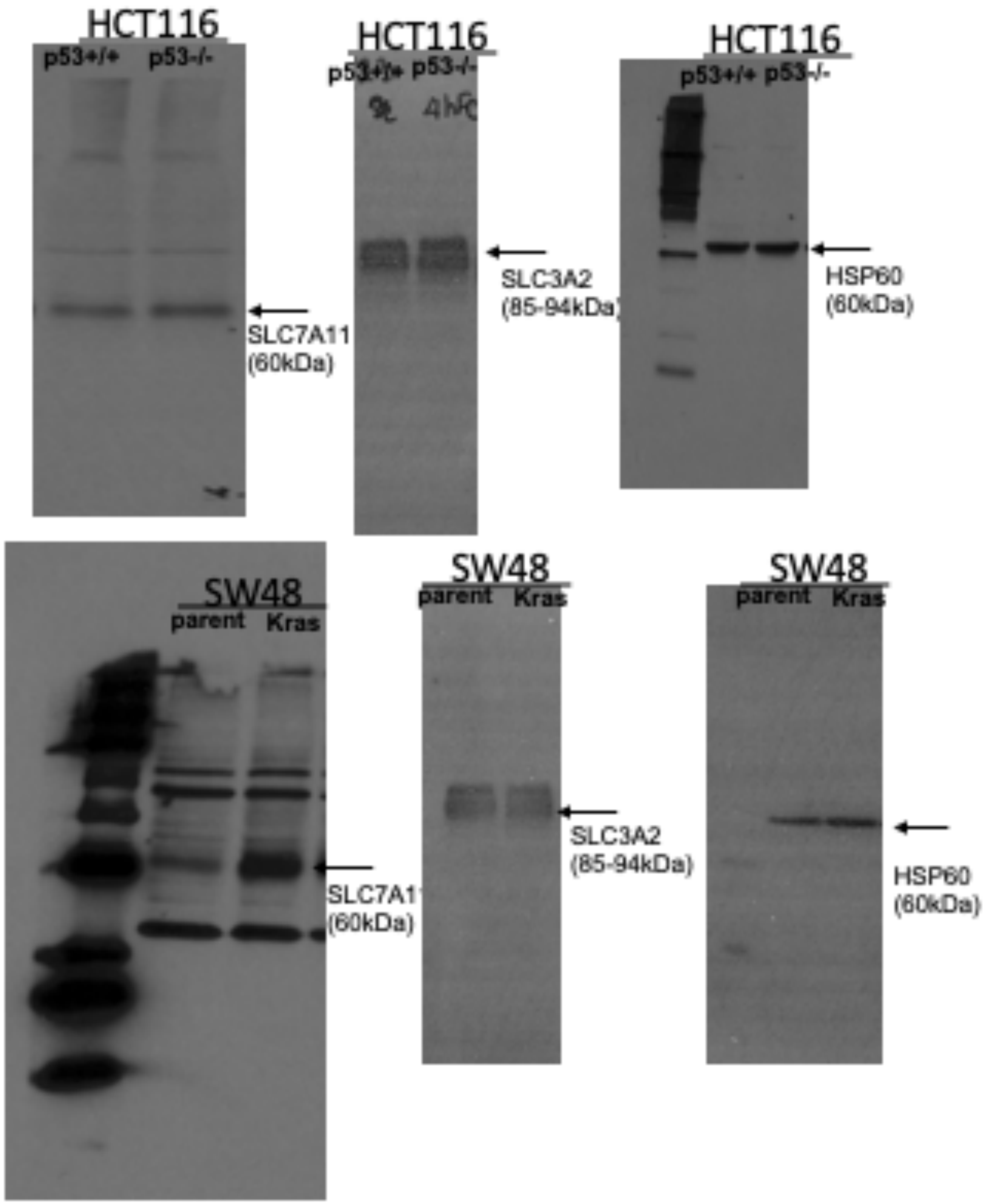

Supplementary Figure S2. Original blot for Fig. 4E & F.

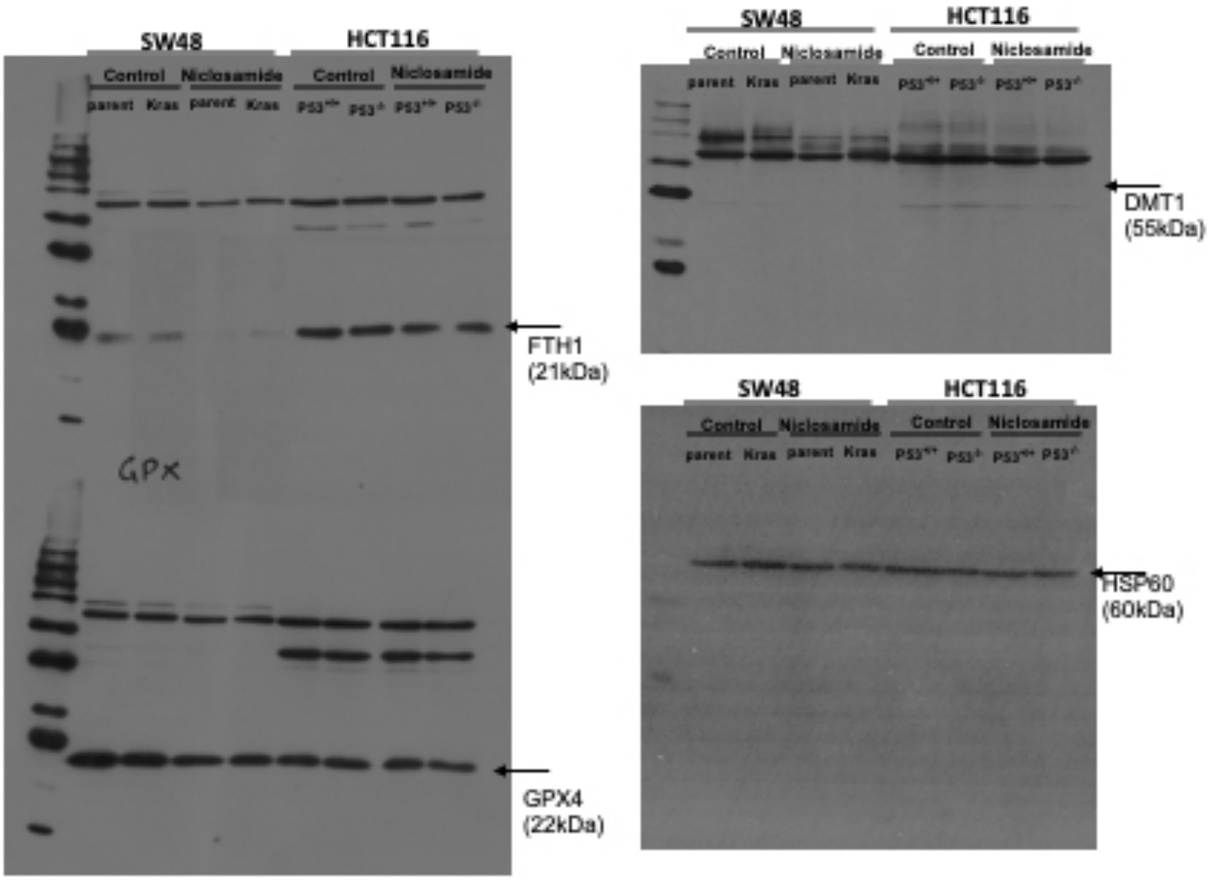

Supplementary Figure S3. Original blot for Fig. 14.
